# A family history of suicide in bipolar disorders: powerful, powerless

**DOI:** 10.1192/j.eurpsy.2023.838

**Published:** 2023-07-19

**Authors:** M. Sagué Vilavella, G. Fico, G. Anmella, A. Giménez, M. Gómez Ramiro, M. T. Pons Cabrera, S. Madero, A. Murru, E. Vieta

**Affiliations:** 1 Deparment of Psychiatry and Psychology, Hospital Clínic, Barcelona; 2 Department of Psychiatry, Hospital Álvaro Cunqueiro, Vigo, Spain

## Abstract

**Introduction:**

When completing the medical record of a patient with bipolar disoder (BD), hardly anything is more impacting than a family history of completed suicide (FHS). In fact, FHS is a main risk factor for personal suicide attempts and death in this population. There are few modifiable protective factors against suicide in BD, such as lithium treatment and absence of substance abuse.

**Objectives:**

We aimed to explore the relationship between a FHS and clinical characteristics in patients with BD. Given the impact that FHS has on the individual and on healthcare professionals, we hypothesized that it would modify behaviors towards a higher prevalence of the modifiable protective factors against suicide, namely more treatment with lithium and less drug addiction.

**Methods:**

This is a cross-sectional study that included all patients with BD that were followed up in a specialised unit between 1998 and 2020. Only subjects with complete information on FHS were retained for the analysis. We assessed sociodemographic and clinical data and described it with measures of frequency, central tendency and dispersion. Differences between subjects with and without FHS were calculated with χ², Fisher’s exact test and Student’s t-test as appropriate. We set the significance level at p≤0.05. All tests were two-tailed.

**Results:**

The sample consisted of 480 subjects with a mean age of 45.9 years (standard deviation 14.4, range 18-88), of which 54.4% (n=261) were women. 69.2% (n=332) had a diagnosis of BD type I and 30.8% (n=148) of BD type II. 77 subjects (16%) had a FHS. Regarding differences between groups, those with relatives who had committed suicide did not show statistically significant differences in terms of sociodemographic variables (age, gender, civil status, employment) or key clinical features (type of BD, illness duration, psychotic features, predominant polarity, rapid cycling, number of lifetime manic and depressive episodes, comorbid personality disorder), neither did they have a higher use of lithium (55.8% vs 59.3%, p=0.572) nor lower substance use disorder (10.9% vs 15.5%, p=0.34). Predictably, people with FHS had a higher prevalence of family history of mental and affective disorders (96.1% vs 70.9%, p<0.001; 86.3% vs 56.3%, p<0.001) and of stressful life events (71.6% vs 58.9%, p=0.05). Personal lifetime suicide attempts also tended to be higher (36.4% vs 26.7%, p=0.088).

**Conclusions:**

Contrary to our hypothesis, in our sample of subjects with BD a FHS was not associated with a higher prevalence of the modifiable protective factors against suicide. Therefore, although suicide has a major impact both in families and healthcare professionals, our results suggest it does not modify attitudes towards prevention in a real-life scenario. The main limitation of our study is its cross-sectional design, which does not allow for causal inference. In conclusion, there is room for improvement in the fight against suicide.

**Disclosure of Interest:**

None Declared

